# Friction Property of Hierarchical Micro/Nanopatterned PDMS

**DOI:** 10.3390/ma15248736

**Published:** 2022-12-07

**Authors:** Gang-Min Kim, Jeong-Won Lee, Sung-Jun Lee, Chang-Lae Kim

**Affiliations:** 1 Korea Automotive Technology Institute, Yeongam-gun 58463, Republic of Korea; 2 Department of Mechanical Engineering, Chosun University, Gwangju 61452, Republic of Korea

**Keywords:** friction, hierarchical micro/nanopattern, PDMS, surface structure

## Abstract

Polydimethylsiloxane (PDMS) has many advantages, but the friction coefficient generated by contact with the counter material is high. The purpose of this study is to reduce the friction coefficient by forming hierarchical micro/nanopatterns on the PDMS surface using the imprinting method. In addition, the optimum conditions for reducing the friction coefficient by controlling the sliding speed and normal load were determined. After contacting flat bare PDMS and hierarchical micro/nanostructured PDMS with a counter tip made of polyurethane (PU), the change in friction with sliding speed and vertical load was evaluated. Under normal load conditions, the average friction coefficient of the bare PDMS decreased as the sliding speed increased, and that of the patterned PDMS slightly increased. Regardless of the sliding speed, the friction coefficient decreased as the normal load increased for both specimens. At a sliding speed of 4 mm/s under a load of 10 mN, the friction reduction effect of the pattern structure was the largest at 79%. Overall, the greatest friction reduction effect (84%) was confirmed in patterned PDMS with the lowest friction coefficient under the conditions of 4 mm/s, 50 mN, compared to bare PDMS with the highest friction coefficient under the conditions of 4 mm/s, 10 mN.

## 1. Introduction

PDMS has high elasticity and rigidity, and is known to be a transparent, flexible, and biocompatible material [1]. Owing to its excellent formability, it is widely used as a material to produce a desired shape or form a pattern on the surface [2]. In addition, it can be used as a biomaterial in the biomedical field and as a substrate for flexible electrodes in transparent/flexible/wearable electrode devices [3,4]. When PDMS is used as a substrate or intermediate material, it is important to have an appropriate adhesive property with the counter material to be bonded. Moreover, when the surface of PDMS coated on a certain material is in direct contact with the moving counter material, the friction and wear properties of the PDMS should be considered. It has been reported that because the PDMS material induces high friction with the mating surface and is a polymer material, the surface of PDMS is severely damaged by the repeated sliding motion of a metal-based tip, which has a relatively high mechanical strength [5,6]. Since PDMS has strong adhesive properties and is a soft/flexible polymer, when it comes into contact with the mating surface, the surface of PDMS is easily press-fitted to increase the contact area, resulting in a large frictional force, which causes severe damage [7].

Various experimental methods have been used to improve the friction properties of PDMS [6,8,9]. As a representative example, the friction property was significantly improved by mixing a PDMS composite with other polymer materials with low-friction properties or solid micro/nanomaterials to reduce the contact area [6]. However, because heterogeneous materials are mixed, the inherent advantages of pure PDMS are weakened, and its chemical and mechanical properties are changed, thereby limiting the field of application. In particular, although the friction and wear properties were greatly improved by mixing microparticles into PDMS, there was a problem in that transparency and flexibility, which are the advantages of PDMS, deteriorated [8]. Accordingly, by manufacturing a composite material in which transparent microspheres are lightly embedded only on the surface of PDMS, the friction and wear properties were improved, while maintaining the transparency and flexibility of the PDMS to some extent [9,10]. Although the transparency and flexibility were improved compared to the case of mixing a large quantity of microspheres into the PDMS, there was a problem in that the microspheres that were only slightly embedded in the surface came out due to the repeated contact sliding motion.

For this reason, to eliminate the disadvantages caused by other materials mixed with PDMS, we attempted to form a specific pattern on the surface of PDMS that could improve the friction properties using only PDMS itself without adding other materials [2]. First, the master surface is made by forming the opposite morphology of the pattern to be shaped on a plane, such as a silicon wafer, aluminum, or stainless-steel plates. A desired surface pattern can be formed on the PDMS surface by the imprinting method, in which the PDMS in solution is poured on the master pattern surface and cured, and then the master is removed. The PDMS surface, on which surface patterns with various shapes such as cylinders and square pillars were formed, improved the friction properties to some extent by reducing the contact area with the counter surface [11]. However, because the surface pattern has a size of several tens to several hundreds of micrometers, the contact area is still large, and in some cases, as the columnar patterns are bent by a sliding motion, a repulsive force acts to increase the frictional force. In addition, complicated and difficult methods such as lithography and laser processing are used to form the micropattern on the master surface, and there is a limitation in that it requires a flat surface.

In this study, a relatively simple etching method that can form random nanoscale morphologies on the surface of a metal plate by immersing it in a chemical solution was utilized. Since the surface of the metal plate is etched by a chemical reaction simply by immersing the metal plate, it is easy to form a pattern on the surface of an object with a complicated shape. In addition, because of the chemical reaction between the atoms of the metal plate and the chemical solution, the size of the pattern formed on the surface to be etched is of a micro/nanoscale, and a very small contact area with the counter surface can be expected. After the PDMS solution poured on the master surface is cured, it is peeled off, and a pattern with random micro/nanoscale morphologies, which is the opposite shape of the master surface, can be formed on the cured PDMS surface. For this hierarchical micro/nanopatterned PDMS, the friction properties were analyzed by changing the sliding speed and normal load.

## 2. Materials and Methods

### 2.1. Materials

**Figure 1:**
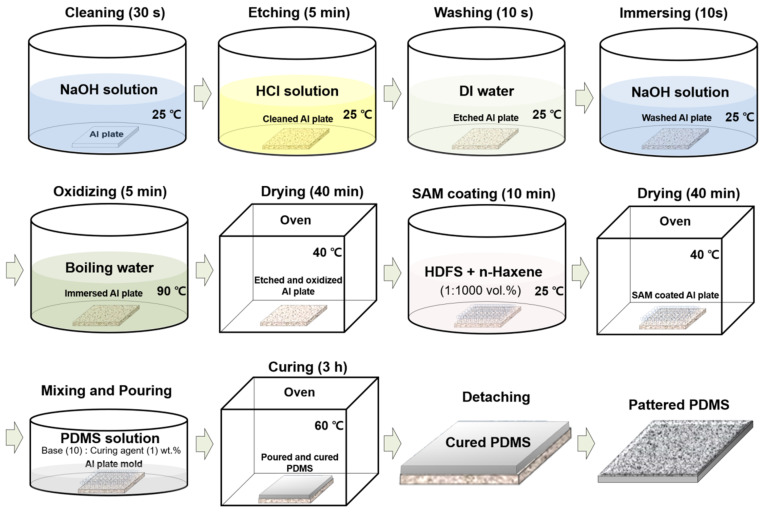
Fabrication process of micro/nanopatterned PDMS.

To form hierarchical micro/nanopatterns on the PDMS surface, an aluminum (Al) plate (Al5052, >97%) was used to fabricate a master for use in the imprinting process. Figure 1 shows the process of fabricating the imprinting master with hierarchical micro/nanopatterns formed on the Al plate and manufacturing PDMS with hierarchical micro/nanopatterns formed by applying PDMS solution to the master and curing it. First, the Al plate was immersed in sodium hydroxide (NaOH, 1 M) solution at 25 °C for 30 s, and then cleaned to remove impurities adhering to the surface. The cleaned Al plate was then immersed in hydrochloric acid (HCl, 3 M) solution and etched at 25 °C for 5 min. The etched Al plate was washed and immersed in deionized (DI) water and NaOH solution at 25 °C for 10 s, respectively. Through this process, random hierarchical micro/nanostructure patterns were formed on the surface of the Al plate. However, because the etched surface exposes pure Al to the air, it is necessary to form a uniform, chemically stable surface over the entire pattern. Accordingly, the etched Al plate was placed in boiling water at 90 °C and subjected to an oxidizing process for 5 min. The surface of the Al plate became stable and corrosion-resistant due to the formation of an oxide film. The Al plate was placed in an oven(LK lab Korea, Namyangju, Korea) and dried at 40 °C for 40 min to remove any liquid remaining within the hierarchical micro/nanostructured patterns formed on the surface. Through this process, the pattern formed on the Al plate surface was oxidized to become chemically stable. However, because the PDMS to be imprinted has a strong adhesive strength, a solution in which heptadecafluoro-1,1,2,2-tetrahydrodecyl-trichlorosilane (HDFS) and *n*-Hexane (C_6_H_14_) were mixed at 1:1000 was used to form a superhydrophobic self-assembled monolayer (SAM) coating on the patterned surface to make it easy to peel off. It was completely dried in an oven at 40 °C for 40 min. The PDMS solution, in which the PDMS base and curing agent were mixed in a weight ratio of 10:1, was poured onto the hierarchical micro/nanopatterned master on which the oxide film and SAM coating were formed, and then cured in an oven at 60 °C for 3 h. The hierarchical micro/nanopatterned PDMS, which has a shape opposite to the surface pattern of the master, was peeled off. To evaluate the effect of hierarchical micro/nanopatterns on the friction properties of PDMS, bare PDMS cured on a flat Al plate was also prepared as a comparative specimen.

### 2.2. Experiments

The surface morphologies of the flat bare PDMS and hierarchical micro/nanopatterned PDMS specimens were measured using a high-resolution field emission scanning electron microscopy (FE-SEM) (FESEM, JEOL, Tokyo, Japan). The surface roughness values of the two specimens were obtained using a 3D laser scanning confocal microscope (3D LSCM, VK-X200, KEYENCE Co., Osaka, Japan).

The friction properties of the flat bare PDMS and hierarchical micro/nanopatterned PDMS specimens were measured using a tribotester (RFW 160, NEOPLUS Co., Ltd., Daejeon, Republic of Korea) driven by a reciprocating sliding motion [12,13,14,15]. Polyurethane (PU) film was attached to a spherical steel ball tip with a diameter of 2 mm to be used as a counter tip. The metal counter tip has a relatively high strength, which causes severe wear on the PDMS specimen [16]. As PDMS is a relatively weak polymer material, if it is used as a counter tip, strong adhesion and severe wear occur, making it difficult to accurately analyze the friction properties [17,18]. Therefore, a PU film that can induce a high frictional force without causing significant damage to the specimen was attached to the surface of the counter tip [19]. Table 1 summarizes the experimental conditions used in this study. When PDMS and PU, which are different polymers, come into contact, the degree of compressive deformation is expected to differ, depending on the normal load. Variations in the friction properties according to the degree of surface compression were compared for normal loads of 10, 30, and 50 mN. In addition, to compare the effects of the elastic deformation and recovery of polymer materials on the friction properties, friction experiments were performed at sliding speeds of 4 mm/s (1 Hz), 12 mm/s (3 Hz), and 20 mm/s (5 Hz). For each experiment, the reciprocating sliding motion was performed for 1000 cycles and repeated 3 or more times under the same experimental conditions to verify the reliability of the experimental results. All experiments were performed at room temperature of 20–25 °C and relative humidity of 30–40%.

## 3. Results

**Figure 2:**
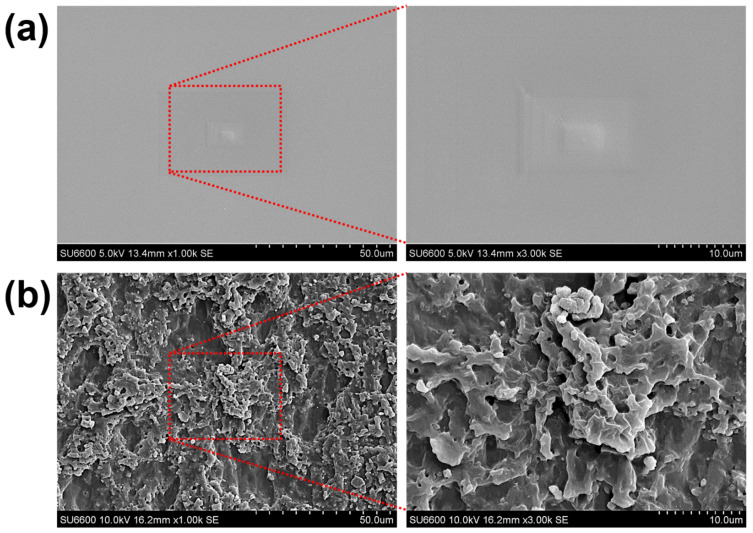
SEM images of surface morphologies of PDMS specimens imprinted on (**a**) flat Al plate and (**b**) micro/nanopatterned Al plate.

Figure 2a,b show the surface morphologies of the PDMS specimens imprinted on a flat Al plate and a hierarchical micro/nanopatterned Al plate, respectively. Figure 2a shows the surface of the bare PDMS with a topography opposite to the surface morphology of the flat Al plate. In contrast, in Figure 2b, the surface of the hierarchical micro/nanopatterned PDMS is relatively rough and shows a randomly varied topography. As the Al plate was etched in HCl solution, the grain traces on its surface disappeared, and hierarchical micro/nanostructures were formed on the flat surface, confirming that the imprinted PDMS had a very rough surface morphology. The surface roughness (Ra) of bare PDMS and patterned PDMS is approximately 1.6 and 5.3 μm, respectively.

**Figure 3:**
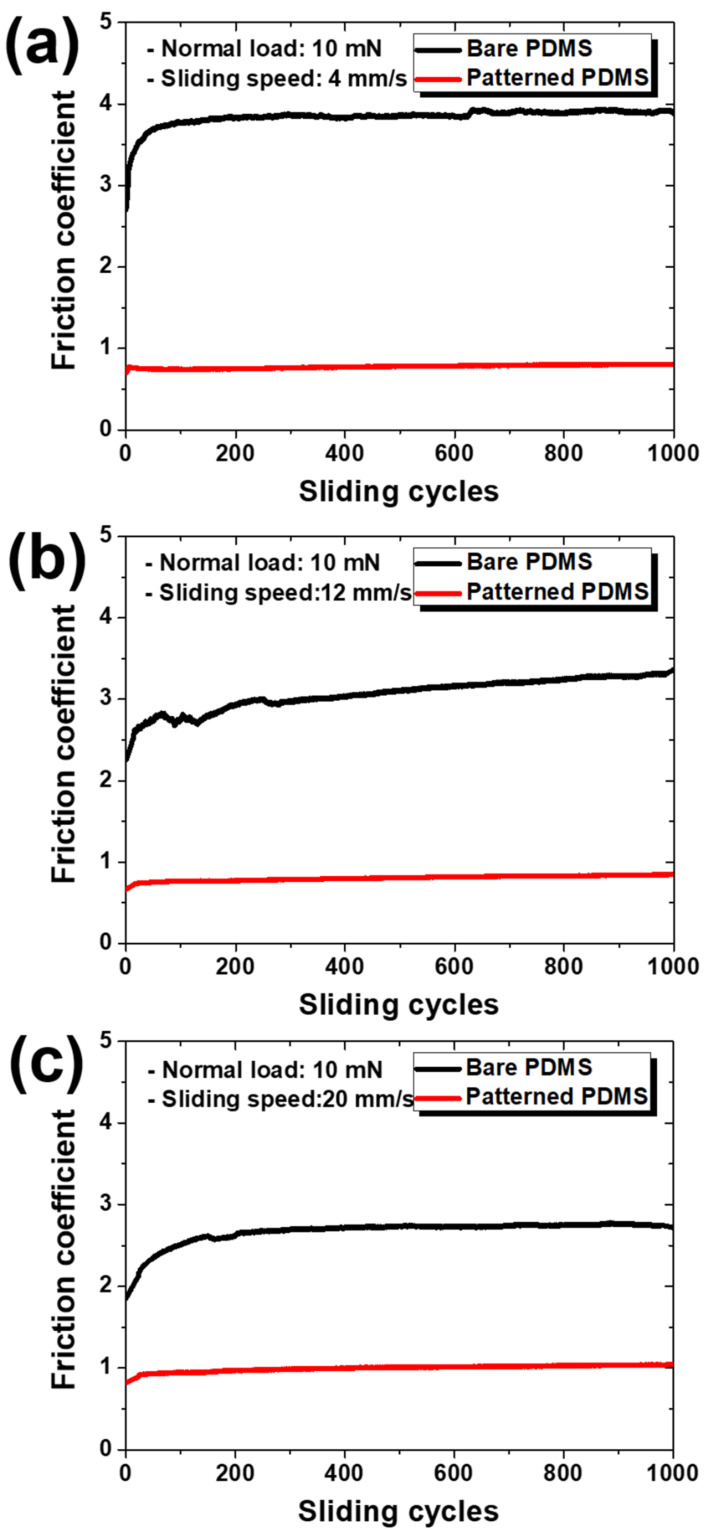
Variations in friction coefficients according to sliding cycles at sliding speeds of (**a**) 4 mm/s, (**b**) 12 mm/s, and (**c**) 20 mm/s under a normal load of 10 mN for bare PDMS and micro/nanopatterned PDMS.

**Figure 4:**
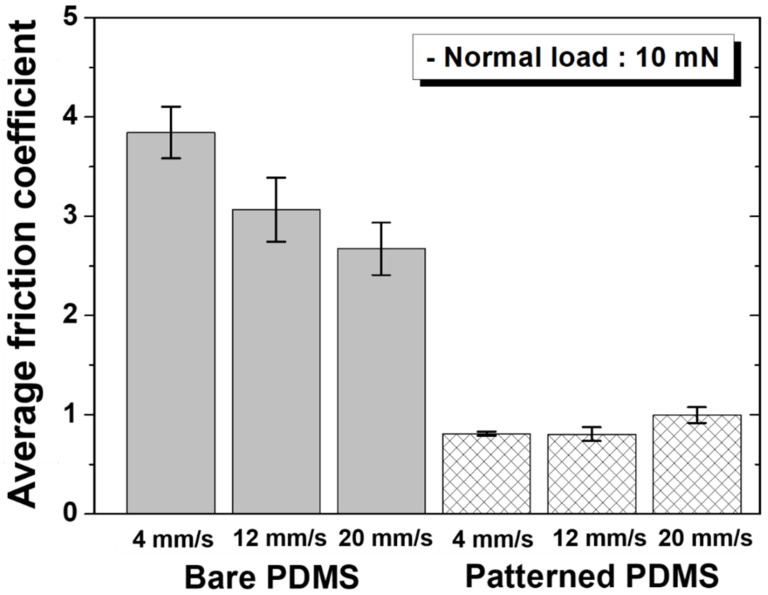
Average friction coefficients as a function of sliding speed under a normal load of 10 mN for bare PDMS and micro/nanopatterned PDMS.

As a result of the friction test conducted for 1000 cycles at sliding speeds of 4, 12, and 20 mm/s under a normal load of 10 mN, the change in the friction coefficient can be confirmed, as shown in Figure 3. Figure 4 shows the average friction coefficient values according to the sliding speed for each specimen. Overall, the friction coefficient of the hierarchical micro/nanopatterned PDMS was significantly lower than that of the bare PDMS. As the number of sliding cycles increased, the initial friction coefficient of the bare PDMS significantly increased, showing a transition period over a long cycle, whereas in the patterned PDMS, the friction coefficient slightly increased in a relatively short period and then saturated over a total of 1000 cycles. As shown in Figure 3, the gap between the friction coefficient values of bare PDMS and patterned PDMS decreased as the sliding speed increased. As shown in Figure 4, as the sliding speed increased, the average friction coefficient of the bare PDMS slightly decreased, whereas that of the patterned PDMS slightly increased. That is, the bare PDMS showed an inverse relationship between the friction coefficient and sliding speed, whereas the patterned PDMS showed a somewhat proportional relationship, but this was a relatively small change. Accordingly, it was considered that the friction coefficient gap between the two specimens decreased as the sliding speed increased. Under a normal load of 10 mN, when the sliding speed was 4 mm/s, which is relatively slow, the decrease in the friction coefficient of the patterned PDMS was the greatest (approximately 79%) compared to that of the bare PDMS.

**Figure 5:**
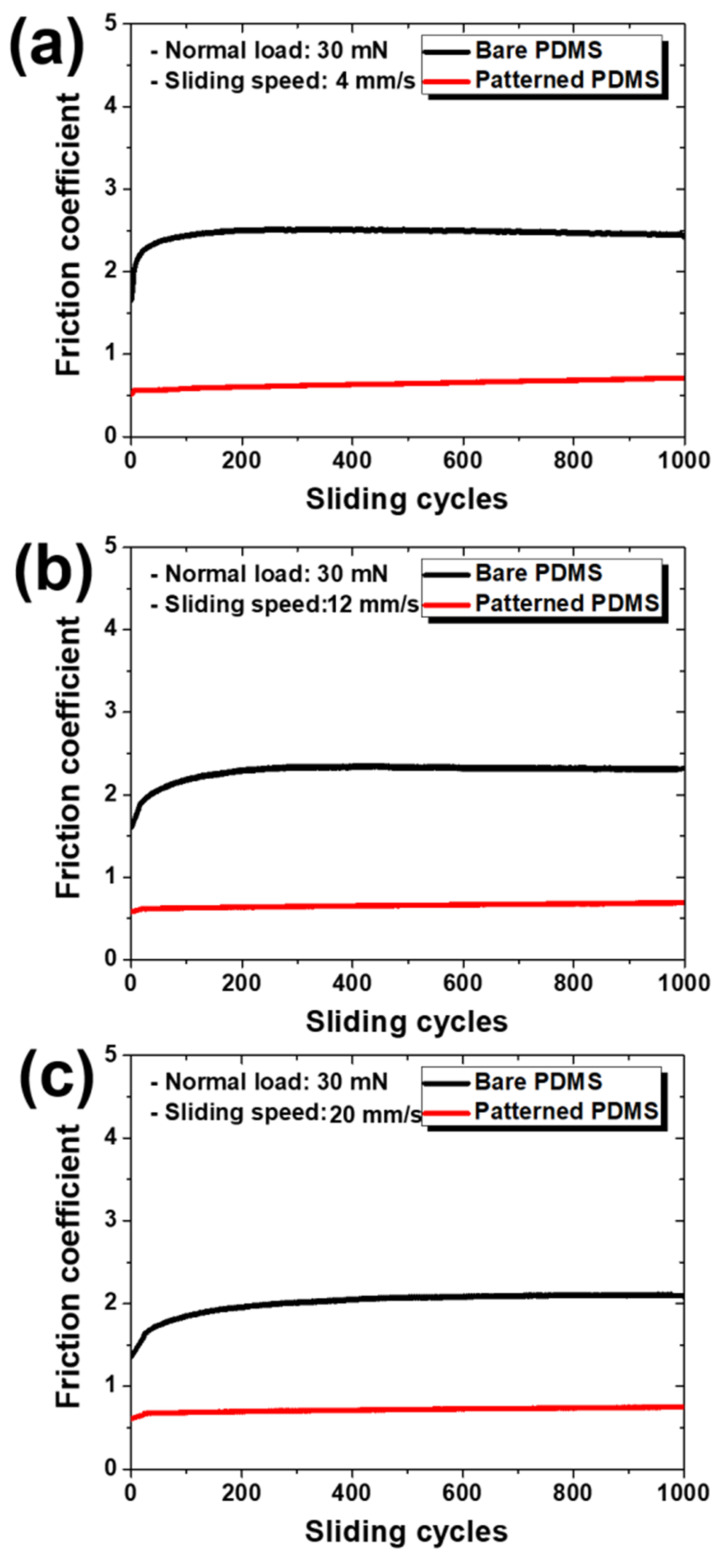
Variations in friction coefficients according to sliding cycles at sliding speeds of (**a**) 4 mm/s, (**b**) 12 mm/s, and (**c**) 20 mm/s under a normal load of 30 mN for bare PDMS and micro/nanopatterned PDMS.

**Figure 6:**
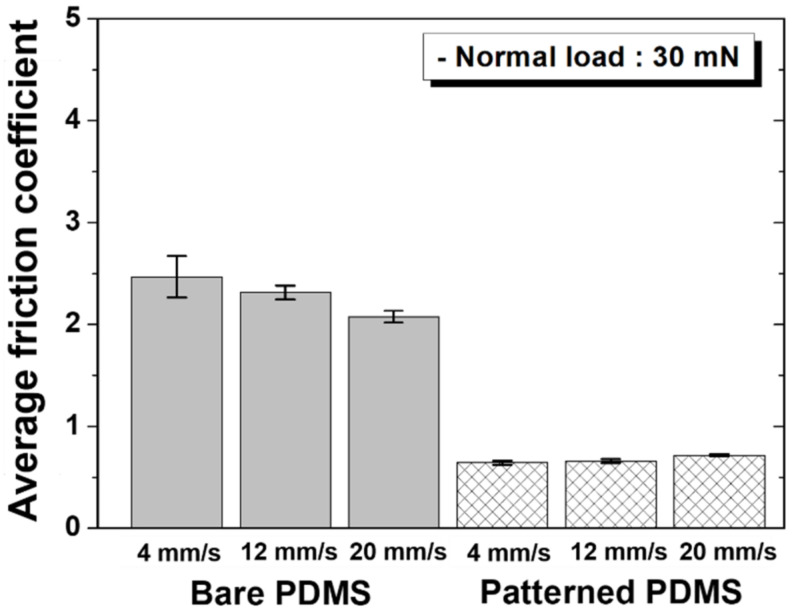
Average friction coefficients as a function of sliding speed under a normal load of 30 mN for bare PDMS and micro/nanopatterned PDMS.

Figure 5 shows the change in the friction coefficient according to the sliding cycle under a normal load of 30 mN for each specimen and sliding speed. As with 10 mN, under a normal load of 30 mN, the bare PDMS also showed a long transition period at the beginning of the sliding cycle, whereas the patterned PDMS showed almost no transition period, and the friction coefficient value at the beginning of the sliding cycle was maintained up to 1000 cycles. The difference between the friction coefficient values of the two specimens also tended to decrease as the sliding speed increased. As shown in Figure 6, this trend appears because the friction coefficient of the bare PDMS decreases and that of the patterned PDMS increases as the sliding speed increases, even at a normal load of 30 mN, as in the case of 10 mN. Therefore, the sliding speed condition showing the largest difference in the friction coefficient between the two specimens was 4 mm/s, and the friction coefficient of the patterned PDMS compared to the bare PDMS was reduced by 74%.

**Figure 7:**
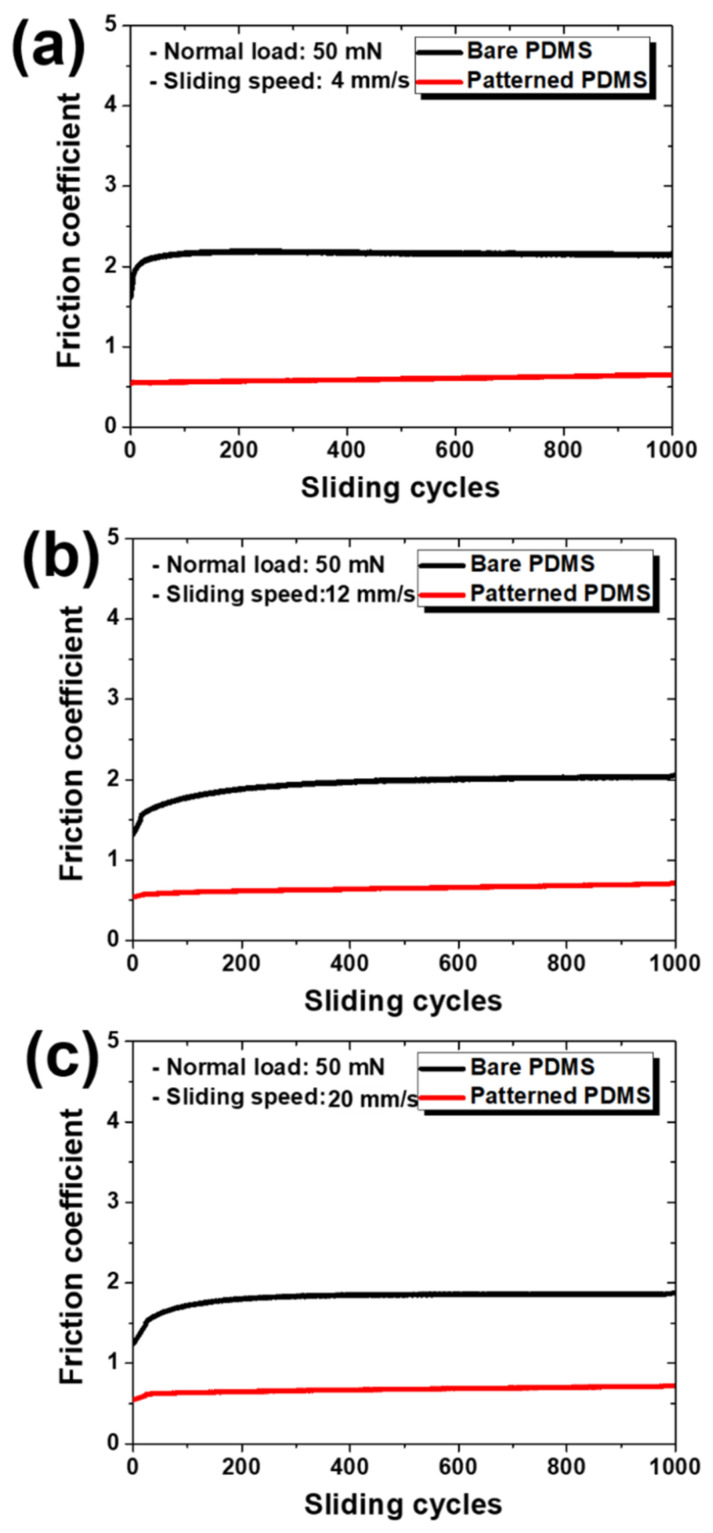
Variations in friction coefficients according to sliding cycles at sliding speeds of (**a**) 4 mm/s, (**b**) 12 mm/s, and (**c**) 20 mm/s under a normal load of 50 mN for bare PDMS and micro/nanopatterned PDMS.

**Figure 8:**
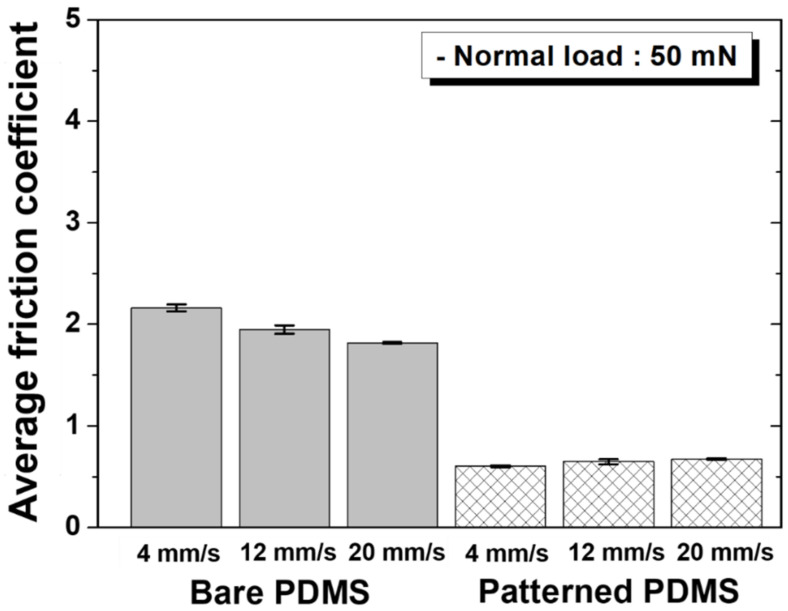
Average friction coefficients as a function of sliding speed under a normal load of 50 mN for bare PDMS and micro/nanopatterned PDMS.

Figure 7 shows the change in the friction coefficient over the entire sliding cycle under a normal load of 50 mN for each specimen and sliding speed. As shown in Figure 8, the friction coefficient of the bare PDMS decreased, and that of the patterned PDMS increased as the sliding speed increased, as in the previous cases. In addition, the difference in the friction coefficient values between the two specimens decreased as the sliding speed increased. Even under a normal load of 50 mN, at a speed of 4 mm/s, which is the slowest sliding speed, the reduction rate of the friction coefficient of the patterned PDMS compared to the bare PDMS was the largest at 72%.

Bare PDMS exhibits a high friction coefficient of 3.84 at a sliding speed of 4 mm/s under a normal load of 10 mN because strong adhesion occurs when it is in contact with the PU film on the counter tip, owing to the strong adhesive properties of the polymer. At a sliding speed of 12 mm/s, the friction coefficient decreased to 3.07, and at 20 mm/s, the friction coefficient was significantly reduced to 2.67. Compared with 4 mm/s, at sliding speeds of 12 and 20 mm/s, the friction coefficient decreased by approximately 20% and 30%, respectively. In the case of normal loads of 30 and 50 mN, the reduction amount of the friction coefficient according to the increase in the sliding speed was smaller than in the case where a normal load of 10 mN was applied. In bare PDMS, under the same normal load, when the sliding speed increased, the adhesion between the PDMS and PU counter tip surfaces was momentarily detached, and the counter tip passed as it was before the compressed surface of the PDMS recovered [5]. Therefore, it is thought that the frictional force between the PDMS and the PU counter tip decreases because the sticking phenomenon that blocks the sliding direction of the tip is weakened. In addition, there is a possibility that the PDMS surface may be softened by the high frictional heat because the time for the frictional heat generated at the two contact surfaces to disappear is relatively short at high sliding speeds [20]. It is believed that the frictional force is reduced because the repulsive force of the PDMS surface acting in the direction opposite to the sliding direction of the counter tip is weakened owing to the softening of the surface. In contrast, in the patterned PDMS, the contact area with the counter tip was greatly reduced because of the hierarchical micro/nanostructure, and the adhesion between the two contact surfaces was reduced, thereby reducing the frictional force. When the sliding motion proceeded at a speed of 4 mm/s under a normal load of 10 mN, the friction coefficient of patterned PDMS was 0.80. When the sliding speed was 12 mm/s, the friction coefficient was 0.80, which was the same as for a sliding speed of 4 mm/s; however, when the sliding speed was 20 mm/s, the friction coefficient was 1.00, an increase of 25%. When the normal load was increased, the rate of increase of the friction coefficient according to the increase in the sliding speed was lower than that of the case with a normal load of 10 mN. It takes a certain amount of time for the hierarchical micro/nanopattern bent in the sliding direction of the counter tip to recover to its original state. When the sliding speed is high, the counter tip comes into contact again before the bent shape of the pattern is restored to its original shape, and the bent or compressed micro/nano-protrusions increase the contact area with the PU surface of the counter tip [21]. Accordingly, because the residual stress remains within the deformed structure, the repulsive force acting in the direction opposite to the sliding of the tip is increased, which is thought to increase the frictional force. However, unlike flat bare PDMS, it is thought that the hierarchical micro/nanopatterned surface of PDMS is less softened because the frictional heat generated by repeated sliding contact with the PU surface is cooled in the space between the pattern structures. It is judged that the non-softened PDMS has a lower friction reduction effect because it maintains its original rigidity. As such, for each normal load, the variation tendency of the friction coefficient values for bare PDMS and patterned PDMS specimens according to the sliding speed was opposite to each other. That is, when the sliding speed was the slowest, the friction coefficient of the bare PDMS was the highest and that of the patterned PDMS was the lowest. From these results, it can be seen that the relationship between the friction coefficient and sliding speed is inversely proportional for the bare PDMS and proportional for the patterned PDMS, regardless of the normal load.

**Figure 9:**
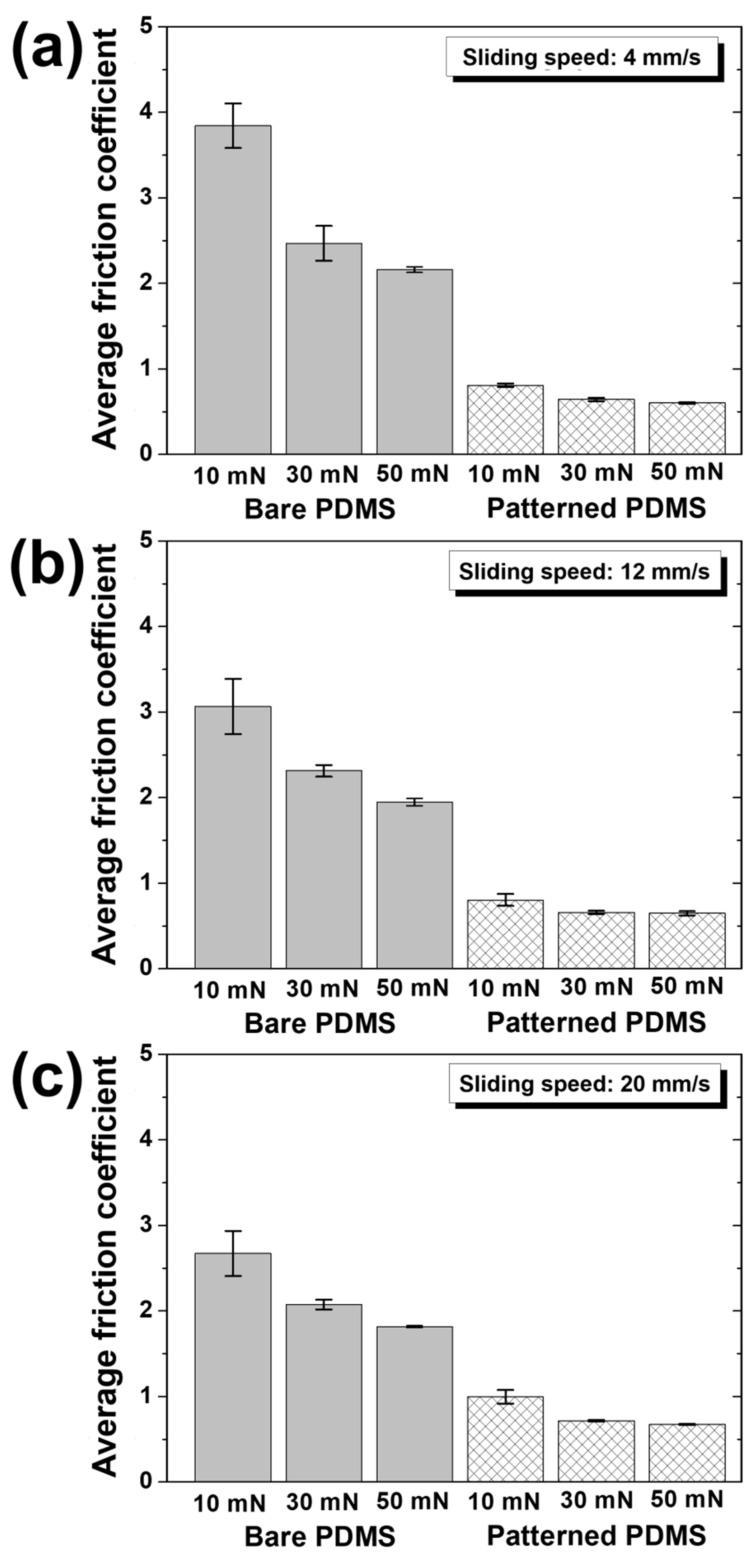
Average friction coefficients as a function of normal load at sliding speeds of (**a**) 4 mm/s, (**b**) 12 mm/s, and (**c**) 20 mm/s for bare PDMS and micro/nanopatterned PDMS.

Figure 9 shows the average values of the friction coefficients of the bare PDMS and patterned PDMS with respect to the normal load for each sliding speed. Regardless of the sliding speed, both bare PDMS and patterned PDMS showed that the average friction coefficient decreased as the normal load increased. Overall, as the normal load increased, the reduction rates of the friction coefficient for the bare PDMS became larger than those for the patterned PDMS. As shown in Figure 9a, at a sliding speed of 4 mm/s, when normal loads of 30 and 50 mN were applied compared to 10 mN, the average friction coefficients of bare PDMS were reduced by 36% and 44%, respectively, and those of patterned PDMS were reduced by 21% and 26%, respectively. In Figure 9b, for a sliding speed of 12 mm/s and normal loads of 30 and 50 mN compared to 10 mN, the friction coefficients of bare PDMS were reduced by 25% and 36%, respectively, and those of patterned PDMS were reduced by 18% and 19%, respectively. In Figure 9c, in the case of 20 mm/s, when the normal load was increased from 10 to 30 mN, the friction coefficients of the bare PDMS and patterned PDMS decreased by 22% and 28%, respectively, and when the normal load was increased to 50 mN, they were decreased by 32% and 33%, respectively. Unlike at sliding speeds of 4 and 12 mm/s, at a sliding speed of 20 mm/s, the reduction rate in the friction coefficient of the patterned PDMS according to the increase in the normal load was larger than that of the bare PDMS. An increase in the normal load means that the hierarchical micro/nanostructures on the patterned PDMS surface are more compressed [22]. When the sliding speed is high, the counter tip passes over the hierarchical micro/nanostructures as they are significantly compressed, so the phenomenon in which the surface of the counter tip is caught by the hierarchical micro/nanostructures is weakened, thereby reducing the friction coefficient [23]. It is thought that the reduction rate of the friction coefficient of the patterned PDMS was larger because the hierarchical micro/nanostructures were compressed more as the normal load increased.

Overall, for bare PDMS, the highest friction coefficient (3.84) was generated when the reciprocating sliding motion was performed at a sliding speed of 4 mm/s under a normal load of 10 mN, and the lowest friction coefficient (1.82) was observed for sliding at 20 mm/s under a normal load of 50 mN. That is, it was confirmed that the friction reduction effect of bare PDMS increased as the sliding speed and normal load increased. The friction coefficients of the patterned PDMS showed the highest value (1.00) at a sliding speed of 20 mm/s under a normal load of 10 mN and the lowest value (0.60) at a sliding speed of 4 mm/s under a normal load of 50 mN. In other words, the friction reduction effect of the patterned PDMS increased as the sliding speed decreased and the normal load increased. Therefore, the lowest friction coefficient (0.60) of the patterned PDMS was for a sliding speed of 4 mm/s and normal load of 50 mN, a reduction by 84% compared to the highest friction coefficient (3.84) of the bare PDMS for a sliding speed of 4 mm/s and normal load of 10 mN.

## 4. Conclusions

In this study, the friction properties of flat bare PDMS and hierarchical micro/nanopatterned PDMS specimens were analyzed. Both specimens were prepared using an imprinting method. Bare PDMS was imprinted by pouring the PDMS solution onto a flat Al plate, and patterned PDMS was imprinted by pouring the PDMS solution onto hierarchical micro/nanopatterns formed on the Al plate surface through surface treatment processes comprising etching, oxidation, and SAM coating. It was confirmed that the surface morphology of the patterned PDMS was rougher than that of bare PDMS. For the two specimens with different surface properties, the changes in friction properties according to the sliding speed (4, 12, and 20 mm/s) and normal loads (10, 30, and 50 mN) were compared. As the sliding speed increased, regardless of the normal load, the friction coefficient of the bare PDMS decreased, while that of the patterned PDMS increased. Under the same experimental conditions, the friction reduction effect of the patterned PDMS was the highest compared to that of the bare PDMS, with a sliding speed of 4 mm/s and a normal load of 10 mN, and the friction coefficient was reduced by 79%. Overall, the friction reduction effect of 84% under the condition of 50 mN, 4 mm/s, showing the lowest friction coefficient in the patterned PDMS compared to the condition of 10 mN, 4 mm/s, showing the highest friction coefficient in the bare PDMS, has appeared. Through this study, it was confirmed that a large reduction in friction can be obtained by controlling the surface patterning and contact conditions (sliding speed and normal load). The results of this study are expected to be helpful in solving the problems in various fields caused by the intrinsic friction properties of PDMS.

## Figures and Tables

**Table 1:** Experimental conditions.

Variable	Condition
Specimen	bare flat PDMS, hierarchical micro/nanopatterned PDMS
Tip (D: diameter)	PU attached steel ball (D: 2 mm)
Normal load	10 mN, 30 mN, 50 mN
Sliding stroke	2 mm
Sliding speed	4 mm/s (1 Hz), 12 mm/s (3 Hz), 20 mm/s (5 Hz)
Sliding cycle	1000 cycles

## Data Availability

Data are available upon request from the corresponding author.
